# Impact of a Conformité Européenne (CE) Certification–Marked Medical Software Sensor on COVID-19 Pandemic Progression Prediction: Register-Based Study Using Machine Learning Methods

**DOI:** 10.2196/35181

**Published:** 2022-03-17

**Authors:** Leevi Limingoja, Kari Antila, Vesa Jormanainen, Joel Röntynen, Vilma Jägerroos, Leena Soininen, Hanna Nordlund, Kristian Vepsäläinen, Risto Kaikkonen, Tea Lallukka

**Affiliations:** 1 Department of Public Health University of Helsinki Helsinki Finland; 2 Solita Oy Tampere Finland; 3 Health and Social Service System Research Welfare State Research and Reform Finnish Institute for Health and Welfare Helsinki Finland; 4 Solita Oy Helsinki Finland; 5 Digifinland Oy Helsinki Finland; 6 IT Centre for Science Helsinki Finland; 7 University of Eastern Finland Kuopio Finland

**Keywords:** health care, health technology assessment, machine learning, COVID-19, COVID-19 forecasting, pandemic, health technology, digital health, online symptom checker, health data, admission data, viral spread

## Abstract

**Background:**

To address the current COVID-19 and any future pandemic, we need robust, real-time, and population-scale collection and analysis of data. Rapid and comprehensive knowledge on the trends in reported symptoms in populations provides an earlier window into the progression of viral spread, and helps to predict the needs and timing of professional health care.

**Objective:**

The objective of this study was to use a Conformité Européenne (CE)-marked medical online symptom checker service, Omaolo, and validate the data against the national demand for COVID-19–related care to predict the pandemic progression in Finland.

**Methods:**

Our data comprised real-time Omaolo COVID-19 symptom checker responses (414,477 in total) and daily admission counts in nationwide inpatient and outpatient registers provided by the Finnish Institute for Health and Welfare from March 16 to June 15, 2020 (the first wave of the pandemic in Finland). The symptom checker responses provide self-triage information input to a medically qualified algorithm that produces a personalized probability of having COVID-19, and provides graded recommendations for further actions. We trained linear regression and extreme gradient boosting (XGBoost) models together with F-score and mutual information feature preselectors to predict the admissions once a week, 1 week in advance.

**Results:**

Our models reached a mean absolute percentage error between 24.2% and 36.4% in predicting the national daily patient admissions. The best result was achieved by combining both Omaolo and historical patient admission counts. Our best predictor was linear regression with mutual information as the feature preselector.

**Conclusions:**

Accurate short-term predictions of COVID-19 patient admissions can be made, and both symptom check questionnaires and daily admissions data contribute to the accuracy of the predictions. Thus, symptom checkers can be used to estimate the progression of the pandemic, which can be considered when predicting the health care burden in a future pandemic.

## Introduction

### Background

The rapid spread of the SARS-CoV-2 virus leading to a pandemic presented challenges for nationwide assessment of the progression of the COVID-19 pandemic [1]. The virus was first discovered in Wuhan, China, in December 2019, and COVID-19 was declared a pandemic by the World Health Organization in March 2020 [2-4]. In Finland, cases started to appear in late February 2020, and the Finnish government announced a national lockdown in mid-March 2020 to slow the viral spread and protect risk groups [1].

Digital health technology tools such as symptom checkers have been used in different countries (eg, Finland, France, Israel, Italy, the Netherlands, the United Kingdom, and the United States) as self-triage tools for possible SARS-CoV-2 infections [5-11]. In Finland, a COVID-19 symptom checker was added to a preexisting national Conformité Européenne (CE)-marked medical symptom checker service, Omaolo [5-12]. The web-based symptom checker provides the user with advice on further actions based on a medically approved algorithm. Although there have been studies about how well symptom checkers perform as clinical tools [13], to our knowledge, the potential of these data for predicting epidemic progression has not yet been studied. Having real-time comprehensive data on reported symptom trends could provide an earlier window into the viral spread and help predict the burden of professional health care.

To study if the data collected by the Omaolo service and the national care notification registers could be used to predict pandemic progression in Finland, we used the methods of machine learning. Perhaps the best potential of machine learning over more traditional methods lies in its ability to better adapt to the data, and thus to the evolution of the underlying phenomenon. With large data sets such as the Omaolo COVID-19 symptom checker responses, machine learning may also uncover more complex associations between the factors contributing to the predicted outcomes. Machine learning models can also be trained and retrained along the way to reveal how the significance of the individual input variables for making the predictions will change over time and to become a more accurate predictor as more data are collected.

### Objectives

The study objective was to assess if a nationwide symptom checker can be used as a predictive tool in estimating the national progression of the COVID-19 pandemic and health care admissions by utilizing machine learning methods.

## Methods

### Data

#### Omaolo

The COVID-19 epidemic in Finland started in mid-March 2020. On March 16, 2020, the Finnish government announced a state of emergency due to the COVID-19 epidemic, and consequently implemented several physical distancing measures aimed at slowing the spread and protecting risk groups [1]. Part of the national response was the Omaolo COVID-19 web-based symptom self-assessment tool, a CE-marked medical device [5,12]. Omaolo was launched for use March 16, 2020, and was published in the two national languages (Finnish and Swedish) and later also in English. The COVID-19 symptom checker functioned as any other symptom checker in Omaolo, and was jointly developed by DigiFinland Oy, Duodecim Publishing Company Ltd, the Finnish Institute for Health and Welfare (THL), Solita Oy, and Mediconsult Oy.

In the symptom checker, the user answers a set of predefined, expert-validated questions. As a result, it returns self-triage information on how to proceed with one’s situation. The progress of filling in the questionnaire from start to finish is recorded to the log files of the service. The respondent has a choice to answer anonymously without including one’s personal information in the process. The questionnaire itself includes several background questions such as age, postal code, gender, and reason for filling in the questionnaire; existing medical conditions; whether the respondent has had close contact with a COVID-19–positive person; whether the respondent or close contacts have been ordered to quarantine by a physician; where the respondent thinks they may have caught the virus; and what kind of work the respondent does in regard to contacts with others (the full Omaolo questionnaire is provided in Multimedia Appendix 1). The questionnaire has been updated several times during the pandemic to better coincide with the latest COVID-19 research [5].

During the study period, a total of 547,428 responses were submitted to Omaolo. Of these, the contents of 132,951 responses were unsaved due to technical reasons. Almost all of the unsaved responses were submitted prior to March 28, 2020, when Omaolo was yet not configured to save the anonymous responses. A small number of the anonymous responses were not saved during short maintenance breaks throughout the period. Accounting for these losses, a total of 414,477 responses were available for analyses. Care reminders, the self-triaged recommendations for care as given by the Duodecim Evidence-Based Medicine Electronic Decision Support service [5], were available for all submitted responses, including the unsaved responses. The data were pseudoanonymized prior to the analyses.

#### National Registers: Hilmo, Avohilmo, and Paavo

We used the established national care notification registries Hilmo and Avohilmo [14] of the THL to estimate the demand of COVID-19–related care. These registers contain structured inpatient (Hilmo) and outpatient (Avohilmo) records from all public and private specialist care hospitals in Finland. These records were combined in the data preprocessing stage and are hereafter referred to collectively as “Hilmo.” The data were anonymized of all identifiers before use.

As a supplementary source, we used the publicly available version of the Paavo register maintained by Statistics Finland [15]. Among other variables, Paavo contains basic demographics of Finnish citizens based on the postal code of their residence. We used these data to identify and rectify the regional bias in age distribution of the Omaolo users. These data were anonymous at the source.

### Predicting the Daily Use of Health Care Resources

We chose our study period to be from March 16, 2020, corresponding to the release of the COVID-19 symptom checker, to June 15, 2020, as the approximate beginning of a period of low activity in the pandemic following the first wave. For the predictions, we used two regressors: linear regression [16] and extreme gradient boosting (XGBoost) regression [17]. The reasoning behind selecting two regressors was to compare a simple and traditional method (linear regression) to a modern option (XGBoost regression) with many hyperparameters that can be learned from the data. Both methods were implemented with three feature preselection strategies: a human expert (KA), F-score [16], and mutual information [18]. All regressor feature selector combinations were tested separately resulting in six different machine learning models.

We chose a scenario where the number of daily COVID-19–related health care admissions, as extracted from the Hilmo register, was predicted 1 week ahead, every week on Wednesdays. This follows a hypothetical scenario where the resources for the following 7 days would be decided midweek (on Wednesday) to give 2 full days to prepare for the weekend, for example by reassessing the need for extra resources and personnel.

For training, testing, and validation of the models, we used time-series nested cross-validation [19]. This strategy was chosen to ensure that the model is trained and tested with samples independent from the validation set; thus, no information from the samples past the prediction point was used. During cross-validation, the set of features (and other hyperparameters in the case of XGBoost), with which the regressor best generalizes its predictions to unseen data in terms of average prediction error on different validation sets, is selected for a given regressor.

We also chose to train the classifiers with Hilmo and Omaolo data first separately and then combined. This was to test how much, if any, the results would improve if the data from both sources were used.

### Profiling the Motives of Omaolo COVID-19 Symptom Checker Users

The Omaolo COVID-19 symptom checker achieved considerable popularity immediately after its release. Tens of thousands of responses per day were submitted during the first week. The submission activity showed clear peaks during infomercials and other major media mentions. To distinguish the users that were truly suspecting a COVID-19 exposure from the users that were visiting Omaolo just out of curiosity, a question about this matter was added to the questionnaire in the form of a simple tick-box on March 28, 2020. After this update, it was found that approximately 40% of the responses had the out-of-curiosity option checked. We then investigated whether it was possible to distinguish the two response profiles (out-of-curiosity or not) and which questions were the best predictors of this behavior. We used a naive Bayes classifier [20], logistic regression [16], and XGBoost binary classifier [17]. We chose naive Bayes and logistic regression because they are widely used in medicine and other fields. XGBoost was included since it is based on a different approach (an ensemble of trees) and thus provides an interesting comparison to the two established methods.

All three models were tested with 5-fold random cross-validation, and the sensitivity and specificity for each fold were computed and finally averaged over all folds. The size of the majority set (not out-of-curiosity) was balanced by undersampling to the size of the minority prior to cross-validation.

All analyses, both the predictions and profiling, were performed using Python version 3.6 [21] with the feature selectors, classifiers, and regressors from Scikit-learn module version 1.0 [22].

### Ethical Consideration

Our study data are based on statistical register data at the national level. These register data contain no personalizing identifiers. Therefore, this study does not fall under the purview of laws regarding medical research. The study protocol does not violate any ethical considerations or standards, according to a statement from the Medical Ethics Committee of the Hospital District of Helsinki and Uusimaa in Finland (June 2013).

## Results

### Predicting the Daily Use of Health Care Resources

Women used the web-based symptom checker more often than men; approximately two-thirds of the filled-in forms were completed by women (Table 1). People of working age were also using the symptom checker more than other age groups. Most of the questionnaires (approximately half) were completed in Southern Finland. Cough was the most common symptom, followed by sore throat, fever, headache, and difficulty breathing.

**Table 1:** Distribution of age, employment status, symptoms, and region of the filled-in Omaolo questionnaires.

Characteristic			Total, n (%)		Men, n (%)		Women, n (%)
**Age (years)**							
	0-9	8652 (2.08)		4291 (2.81)		4361 (1.66)	
	10-19	30,805 (7.41)		10,689 (6.99)		20,116 (7.68)	
	20-29	93,511 (22.50)		30,241 (19.77)		63,270 (24.10)	
	30-39	98,041 (23.59)		34,727 (22.71)		63,314 (24.11)	
	40-49	77,869 (18.74)		29,010 (18.97)		48,859 (18.61)	
	50-59	59,432 (14.30)		22,788 (14.90)		36,644 (13.96)	
	60-69	31,935 (7.69)		13,392 (8.76)		18,543 (7.06)	
	70-79	12,824 (3.09)		6519 (4.26)		6305 (2.40)	
	≥80	2453 (0.59)		1286 (0.84)		1167 (0.44)	
	Total	415,522		152,943		262,579	
**Employment status**							
	Not currently working	133,199 (32.59)		47,674 (31.90)		85,525 (32.99)	
	Health care worker	65,379 (16.00)		7953 (5.32)		57,426 (22.15)	
	Cannot avoid contact (service worker)	102,544 (25.09)		41,948 (28.07)		60,596 (23.37)	
	Can avoid contact	107,558 (26.32)		51,869 (34.71)		55,689 (21.48)	
	Total	408,680		149,444		259,236	
**Symptoms**							
	Cough	140,661 (34.60)		53,364 (36.53)		87,297 (33.51)	
	Trouble breathing	31,143 (7.66)		11,312 (7.74)		19,831 (7.61)	
	Sore throat	86,985 (21)		24,505 (16.78)		62,480 (23.99)	
	Headache	35,886 (8.83)		12,233 (8.37)		23,653 (9.08)	
	Myalgia	10,213 (2.51)		4552 (3.12)		5661 (2.17)	
	Vomiting	7406 (1.82)		2640 (1.81)		4766 (1.83)	
	Fever	59,041 (14.52)		23,842 (16.32)		35,199 (13.51)	
	Loss of smell	433 (0.11)		182 (0.12)		251 (0.10)	
	Dysphagia	7971 (1.96)		2946 (2.02)		5025 (1.93)	
	Trismus	6469 (1.59)		1938 (1.33)		4531 (1.74)	
	Trouble speaking	6392 (1.57)		2971 (2.03)		3421 (1.31)	
	Other	13,952 (3.43)		5589 (3.83)		8363 (3.21)	
	Total	406,552		146,074		260,478	
**Region**							
	Western central Finland (city of Tampere)	42,759 (14.50)		15,370 (14.41)		27,389 (14.55)	
	Western coastal Finland (city of Turku)	36,321 (12.31)		13,155 (12.33)		23,166 (12.30)	
	Northern Finland (city of Oulu)	27,419 (9.30)		9894 (9.27)		17,525 (9.31)	
	Southern Finland (city of Helsinki)	149,145 (50.56)		54,447 (51.04)		94,698 (50.29)	
	Eastern Finland (city of Kuopio)	39,340 (13.34)		13,819 (12.95)		25,521 (13.55)	
	Total	294,984		106,685		188,299	

For the analyses, we chose two regressors, linear regression and XGBoost, with three feature preselection strategies for each, and compared their performance. To predict the COVID-19–related admissions for each day, 7 days ahead of the prediction point, the features given to the model were extracted from the responses and the Hilmo register on the 7 and 14 days prior (lag variables). The use of lag variables essentially means that two sets of the time-dependent features were formed, with the first delayed 7 days and the second delayed 14 days. This was to ensure that no data from any of the sources, Omaolo or Hilmo, were leaked past the point of prediction during feature selection, model training, or testing. The regressors were first trained with 5 expert-selected features: how many of the questionnaires were filled-in by people over 60 years old, how many reported lengths of symptoms were greater than 10 days, and how many were assigned the urgency code P1 (the most urgent) in the care recommendations, in addition to the number of COVID-19–related admissions. The feature preselectors F-score and mutual information were added later.

**Figure 1:**
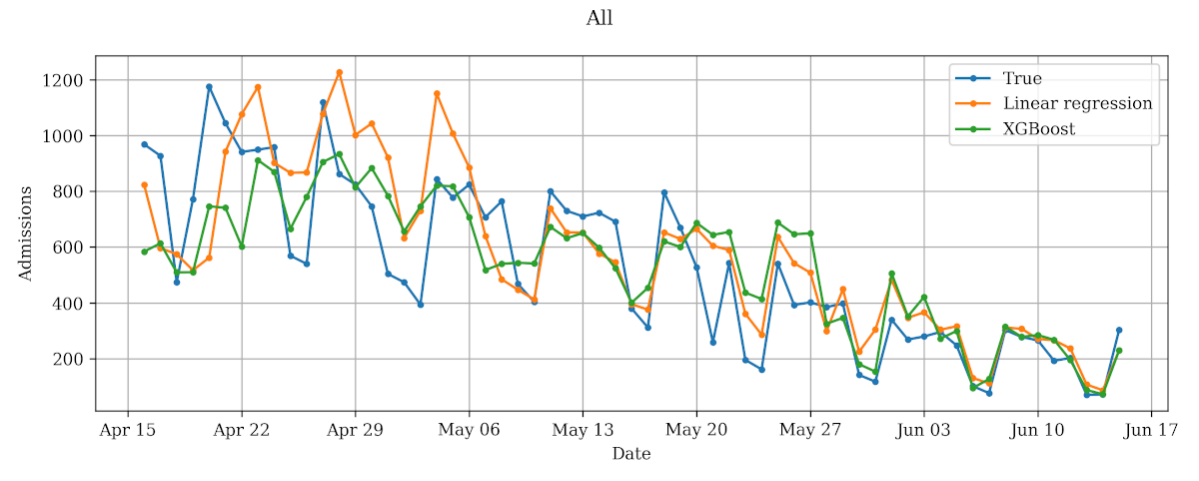
COVID-19–related admissions predicted by linear regression and extreme gradient boosting (XGBoost) regression models, together with the true admission count during the first wave of the pandemic in 2020.

The predictions made by both models for data gathered between March 16, 2020, and June 15, 2020, were compared to the true admission count (Figure 1). The first 4 weeks were reserved for training the models before predictions were made in the week starting on April 16, 2020. For the consecutive weeks, the models were retrained with the data from the previous weeks. Both models made predictions with a similar error (linear regression: mean absolute error [MAE] 138, mean absolute percentage error [MAPE] 31; XGBoost regression: MAE 135, MAPE 31). The predictions become more accurate toward the end of the study period, since the models had more data to learn from, which reduced the sampling bias. May 22, 2020, was a public holiday in Finland, explaining a similar drop in the true admission count as on weekends.

To check that the Omaolo questionnaire data are relevant for the predictions, we compared the error of both models with and without the questionnaire data, and only with the questionnaire data (Table 2). Both models achieved the lowest error when the registry and the questionnaire data were combined, indicating that the questionnaire data are relevant for making accurate predictions compared to the registry data alone.

In addition to expert-selected features, we tested automated feature selection methods that select the top 8 features based on the F-score or mutual information of the feature with the predicted variable (number of admissions on a given day) (Table 3). The F-score and mutual information are measures of dependence between the feature values and the values of the predicted variable in the historical data. Different feature selection strategies worked better for the two models: linear regression was the most accurate with the mutual information criterion, whereas XGBoost was the most accurate with the expert-selected features.

Older age groups, who are more likely to have a severe form of COVID-19 and hence be admitted to hospital, are underrepresented in the Omaolo questionnaire data. This could affect the performance of the models, as it is more difficult for the models to learn from imbalanced training data. To assess the problem, oversampling of the underrepresented age groups was performed to see if it would decrease the error of the models (Table 4). Resampling gave the linear regression model slightly smaller error, whereas the XGBoost regressor performed worse with resampling. These results indicate that it is not essential when minimizing the prediction error to oversample the questionnaire answers of the underrepresented age groups to match the age distribution of the population.

**Table 2:** Comparison of the effect of Omaolo^a^ and Hilmo^b^ data on the error of the models on expert-selected features.

Features		Mean absolute error	Mean absolute percentage error
**Linear regression**			
	Omaolo+Hilmo	137.79	31.33
	Omaolo	175.38	44.60
	Hilmo	184.37	34.98
**Extreme gradient boosting regression**			
	Omaolo+Hilmo	139.11	31.78
	Omaolo	165.18	46.94
	Hilmo	178.92	46.63

^a^Omaolo: A web-based, CE-marked symptom self-assessment tool and medical device.

^b^Hilmo: National administrative register on hospital admissions.

**Table 3:** Comparison of the effect of different feature selection methods on the error of the models.

Feature selection		Mean absolute error	Mean absolute percentage error
**Linear regression**			
	Expert-selected	137.79	31.33
	F-score	141.40	30.46
	Mutual information	112.16	24.23
**Extreme gradient boosting regression**			
	Expert-selected	139.11	31.78
	F-score	150.07	36.40
	Mutual information	146.12	33.33

**Table 4:** The effect of oversampling on the error of the models with expert-selected features.

Resampling			Mean absolute error		Mean absolute percentage error
**Linear regression**					
	No resampling	137.79		31.33	
	Oversampling	131.13		30.47	
**Extreme gradient boosting regression**					
	No resampling	139.11		31.78	
	Oversampling	153.35		35.22	

### Profiling the Motives of the Omaolo COVID-19 Symptom Checker Users

The three different models produced the following sensitivity and specificity for detecting users that answered they were using the service out of curiosity: 0.622 and 0.367, respectively, for the naive Bayes classifier; 0.665 and 0.332, respectively, for logistic regression; and 0.607 and 0.388, respectively, for the XGBoost binary classifier. These results were acquired by maximizing the number of correct classifications.

## Discussion

### Principal Results

In this study, we examined whether it is possible to predict the national epidemic progression and the burden of health care using machine learning methods with real-life data on symptoms and usage of health care. The main finding of this study is that it is possible to predict national health care admissions related to COVID-19 using a symptom checker combined with register data by using machine learning methods with considerable accuracy (small MAPE error). These methods were tested in a scenario where the predictions were made 1 week ahead, once per week. The best model was achieved using the symptom checker data combined with register data (MAPE 24.23%). This result was reached by using linear regression with mutual information as the feature preselector. All tested models and combinations of feature preselectors and models were able to produce predictions that followed the true epidemic progression (Figure 1). Overall, linear regression was better than XGBoost, although only marginally. This suggests that in our research scenario, there was no benefit in using a model that has many trainable hyperparameters (XGBoost regression) over a simple model (linear regression). All tested models seemed to improve toward the end of our study period as more data were available for training. Additionally, the differences in accuracy between the models were more visible at the start of the period and seemed to diminish toward the end. Based on the results, the F-score and especially mutual information appear to improve the results for linear regression. Feature preselection may improve the predictions by, for example, reducing the risk of overfitting. This is relevant in our data set since the feature set used in the classification was rather large and likely suffers from multicollinearity. Using the preselectors did not improve XGBoost regression. This suggests that we were not able to find a suitable preselection strategy for the method.

Finally, predictions that can follow the progression can be made using either Omaolo symptom checks or historic Hilmo counts separately. However, the best results were achieved by combining both. Adding Omaolo to Hilmo counts reduced the MAPE of linear regression from 34.98% to 31.33% and that of XGBoost from 46.63% to 31.78%. These results suggest that Omaolo contains information of the pandemic progression that is not present in Hilmo alone.

Oversampling the data to balance the regional differences between the Omaolo users and general population seemed to produce conflicting results: marginal gain with linear regression but loss of accuracy with XGBoost. Oversampling leads to added complexity in the analysis pipeline, and without a clear benefit, its use is hard to justify.

### Profiling the Motives of the Omaolo COVID-19 Symptom Checker Users

The answer profiles of those using the COVID-19 symptom checker out of curiosity were very similar to those of the other users, and no reliable classification between the groups could be made by any of the tested models. Neither of the groups reported longer or more serious symptoms over the other. The only striking difference between the groups was that there appeared to be more out-of-curiosity responses during high service utilization such as after television infomercials.

### Consideration of Other Sources

We also considered using the daily number of phone calls received at the 116117 Medical Helpline service. The 116117 Medical Helpline provides professional assistance on health care–related topics in urgent, but nonemergency cases to over 4 million Finns in extended business hours. However, since it was not possible to extract the topic of the call, whether they were COVID-19–related or not, and since the calls could be localized with much poorer resolution than with the rest of the sources, this data set was eventually dropped from the analyses.

The Google Trends [23] of popular COVID-19–related search terms was another potential data source considered for analyses. However, it was found that the publicly available Finnish trends only covered major cities. Additionally, the overlap of trending search terms between cities was found to be small, making the data very sparse. For these reasons, this data set was not used in the analyses.

### Strengths and Limitations

This study had several potential limitations. A considerable number of responses (132,951) were not saved during the study period. Nearly all of these were submitted during the first 2 weeks of the study, complicating the analysis for the first month. This may have contributed to the relatively poor prediction accuracy for the related weeks by delaying the convergence of the regressors to the true admission count.

The true admission count showed a strong diminishing trend toward the end of our study period. During the last weeks of the period, there were days when only a few dozen new admissions were recorded nationally. Because our error metric, MAPE, is relative to the true values, if these values are small, error values will appear high even though the absolute error between true and predicted counts remains low. Despite this, we decided to use MAPE for its intuitiveness, wide use, and easy comparability of the error between the days, weeks, and methods.

At the beginning, the survey did not include an item about the motive to fill in the survey (ie, whether it was due to actual symptoms or out of curiosity). This adds some additional forms to our data that do not reflect the situation at hand. The proportion of responses filled-in out of curiosity remained remarkably stable at around 40% throughout the study period. Moreover, the results of trying to separate the responses filled-in out of curiosity from the rest with binary classifiers (naive Bayes, logistic regression, XGBoost classifier) failed to reveal any meaningful differences between the answer profiles of these groups. Thus, we did not find a justification to remove these responses from the analyses or handle them differently than the rest of responses (not filled-in out of curiosity).

The data available for this study contained COVID-19–related admissions data with a steady downward trend, and it would have been interesting to see if the models could predict a reversal of the trend before it occurs. However, there is weekly variability in the admissions and the models learned this pattern well. An interesting deviation from the weekly pattern was Ascension Day on Thursday, May 21, which was a public holiday in Finland. The models did not have enough training data on admissions on public holidays on a weekday to predict a similar dip in the admissions as on weekends, although longer data sets could allow the models to learn this pattern as well.

Much of the prediction errors took place during days that showed sharp peaks of increased or decreased demand that were not immediately explainable with the data available. Some of these errors may be due to technical reasons such as a major care provider suffering an error on one day and reporting higher counts on the following day. Naturally, these kinds of special events cannot be learned from the admission count data alone. On a positive note, the developed models appear robust and thus not susceptible to these kinds of anomalies.

One could also question if biological tests make other monitoring redundant. While in many countries biological tests are performed to follow the pandemic, it is important to note that online symptom checking does not replace the need for biological testing but provides a different and valuable perspective. In many countries, including Finland, the testing services are saturated, and only some population groups get tested, making our picture of the pandemic progression biased. Biological testing further comes with an immense cost, particularly with an exponential rise in cases.

The symptom checker, in turn, can be filled in anytime and by large numbers. Furthermore, it can be continuously updated to include the most relevant questions. It is possible that the health care burden is going to change even quite rapidly, and we can see that using Omaolo surveys, even without an indication from the time-series data. Additionally, the machine learning models are trained every week, allowing adaptability to changes in the statistical relationship between the predictors and the predicted variable as the pandemic progresses.

Furthermore, while our data only cover the first wave of the pandemic, the results remain important from the perspective of early decision-making against a new threat, and overcoming the challenge on modeling a novel phenomenon from the start with no history. Additionally, we do not only validate the data, but our approach further enables the prediction and modeling of the pandemic. Emergence of vaccinations, new variants, new policies, and restrictions will all affect the progression of the pandemic. These changes have also affected Omaolo, which has been continuously updated (eg, including questions about vaccination status). Our prediction models are also upgradeable continuously; thus, the drift in data and concept can be mitigated on the go, and features that will drop or rise in importance can be monitored along with the actual result of the prediction.

Finally, the lack of reported anosmia could suggest a lack of specificity. In our data, anosmia is a rarely reported symptom. It is also a known problem that without a specific test, COVID-19 is notoriously hard to distinguish from a common flu. Despite the relatively small number of reported anosmia cases, we have other features that have been shown to be highly significant in predicting International Classification of Diseases-10– and International Classification of Primary Care-2–coded admissions. These include features such as how many of the questionnaires were filled-in by people over 60 years old, how many reported lengths of symptoms were greater than 10 days, and how many were assigned an urgency code P1 (the most urgent) in the care recommendations.

Alternatively, if anosmia is important for specificity, it is likely that we have more false positives and our final models are less accurate. In other words, with the key symptoms, the precision of the model is improved and the error is smaller. The consequence of such a lack depends on how the results are used. If the burden of health care were solely estimated based on these models, we might underestimate the need for health care. However, it is unlikely that the model would be used to define any absolute health care need, whereas it can provide an indication to prepare for an increased health care burden.

The study also has several strengths. We had access to a nationwide online symptom checker data source, Omaolo*,* which is a CE-marked medical device complying with the Medical Device Directive, used by health care and social service professionals [24]. We also had data on all hospital admission records from public or private hospitals in Finland on a weekly basis. Such data are rarely available anywhere in the world and provide unique opportunities to produce new information about the possibilities of using such real-life data in predicting a subsequent health care burden. Similar symptom checkers could be adopted for use in many other countries, and they could provide an opportunity to collect data on symptom development very rapidly and at a relatively low cost at a national level. These symptom checkers and the findings are not restricted or solely applicable to the current pandemic or its first wave, but could be applied in any other future epidemic or pandemic or for the collection of other types of symptoms as well.

In addition, a clear advantage of machine learning methods is that both the model section and fit are automatized. This means that the prediction method adapts in the face of any new data and attempts to make as accurate predictions as possible with the data in use at any given time. However, in case the phenomenon in question changes notably, changes in data use and sources and machine learning methods are also required.

### Conclusions

Our study shows that COVID-19–related health care admissions in the short term can be predicted with considerable accuracy using symptom checker data combined with register data based on machine learning methods. This type of approach could help health care providers better assess the burden of the health care system in advance, which would make resource allocation more predictable. Furthermore, we consider that this type of approach could also be implemented in different stages of the pandemic and in future pandemics as well.

## Appendix

Multimedia Appendix 1 Symptom checker questionnaire.

## Abbreviations

CE: Conformité Européenne
MAE: mean absolute error
MAPE: mean absolute percentage error
THL: Finnish Institute for Health and Welfare
XGBoost: extreme gradient boosting
