# Supplementary material for: Impact of a Conformité Européenne (CE) Certification–Marked Medical Software Sensor on COVID-19 Pandemic Progression Prediction: Register-Based Study Using Machine Learning Methods
Source: JMIR Form Res. 2022 Mar 17;6(3):e35181. doi: 10.2196/35181 (PMC8972109; doi:10.2196/35181)
Supplement: Multimedia Appendix 1 [file formative_v6i3e35181_app1.docx]

**Multimedia Appendix 1. Symptom checker questionnaire.**

©Omaolo symptom check questions July 2020

- I just want to try or test the symptom checker

- What is your postcode (zip code)?

- Your age (in years)

- Gender (answer required for further questions)
  - Male
  - Female

- Are you pregnant?
  - Yes
  - No
  - Not sure

- Why are you filling out this form?
  - I have symptoms and want to know if they are caused by the coronavirus.
    - What symptoms do you have?
      - Cough
      - Difficulty of breathing
      - Sore throat
      - Runny nose or stuffiness (does not mean chronic or allergic symptoms)
      - Headache
      - Muscle pain
      - Diarrhea
      - Vomiting (throwing up)
      - I have lost my sense of smell or taste
      - Other symptoms
    - How many days have your current symptoms lasted? Give the answer as a whole number (for example 4)
    - Do you have fever? (Above 37,5 degrees Celsius?)
      - Yes
        - What has been the highest fever reading you have had in the last 24 hours? Give the answer with a decimal point (for example 37,5 degrees Celsius).
      - No
    - What is your general condition?
      - I am well enough to be out of bed and can complete regular household tasks
      - I am well enough to be out of bed, but I can only complete the most essential household tasks
      - I feel very weak and can only lie in bed (at most I can visit the bathroom)
    - What is your weight (kg)?
    - What is your height (cm)?
    - Do you smoke on a daily basis?
    - Do you have factors that increase your risk of thrombosis (blood clot)? You can select more than one option.
      - A history of deep venous thrombosis (blood clot) or pulmonary embolism
      - Increased hereditary risk for thrombosis (blood clot) that has been verified by a blood test.
      - Active cancer with ongoing treatment
      - Blood or bone marrow disease such as myeloma, polycythemia vera, thrombocytosis, PNH
      - Severe inflammatory disease such as rheumatoid disease, inflammatory bowel disease or vasculitis
      - Major surgical operation, such as knee or hip replacement within 2 months
      - Cast in the lower extremity
      - Delivery (childbirth) within 6 weeks
      - Poorly controlled diabetes
      - Severe renal insufficiency
      - None of the above
    - Please answer the following questions to identify serious symptoms not relating to coronavirus
      - Do you have difficulty of swallowing food or drink because it's so painful?
        - Yes
        - No
      - Do you have difficulty of opening your mouth (lockjaw)?
        - Yes
        - No
      - Have your speech become difficult for others to understand?
        - Yes
        - No
  - I do not have any symptoms but would like to know if I have caught coronavirus.

- During the last 14 days, have you been in the same room or within a 1-meter distance of someone who you know has coronavirus?
  - Yes
  - No

- Have you been ordered into quarantine by a doctor?
  - Yes
  - No

- Where do you think you might have caught coronavirus?
  - I have been abroad in the past 14 days
  - I think I caught it some other way
  - None of the above

- What kind of work do you do?
  - I am not working
  - I work in health care or social services
    - Have you cared for any coronavirus infected patients?
      - No, I do not think so
      - Yes and I had good protection (masks, gloves etc.)
      - Yes and I did NOT have good protection (masks, gloves etc.)
  - I work in a service job where I meet with customers or have close contact with co-workers
  - I work in a job where I can avoid close contact with customers and co-workers

- Do you have an illness, a treatment or other reason that you might have a high risk for severe coronavirus disease?
  - Yes
    - Which illnesses, treatments or conditions do you have that lower your immunity to coronavirus?
  - No

- Do you need help with worry or anxiety caused by the virus or following the news on the epidemic?
  - Yes
  - No

- If medical professional recommend that you should be examined, how would you be able to travel?
  - I would be able to travel alone or with my family member living with me (my own car or other transportation) and so that I would not infect others.
  - I would have to use public transportation.
  - I would have to use a taxi.
  - I am so unwell or it is so difficult for me to walk that I would need an ambulance, medical taxi or a home visit by a nurse.
